# Supplementary figures and images for: Cropland Mapping Using Sentinel-1 Data in the Southern Part of the Russian Far East
Source: Sensors (Basel). 2023 Sep 15;23(18):7902. doi: 10.3390/s23187902 (PMC10536219; doi:10.3390/s23187902)

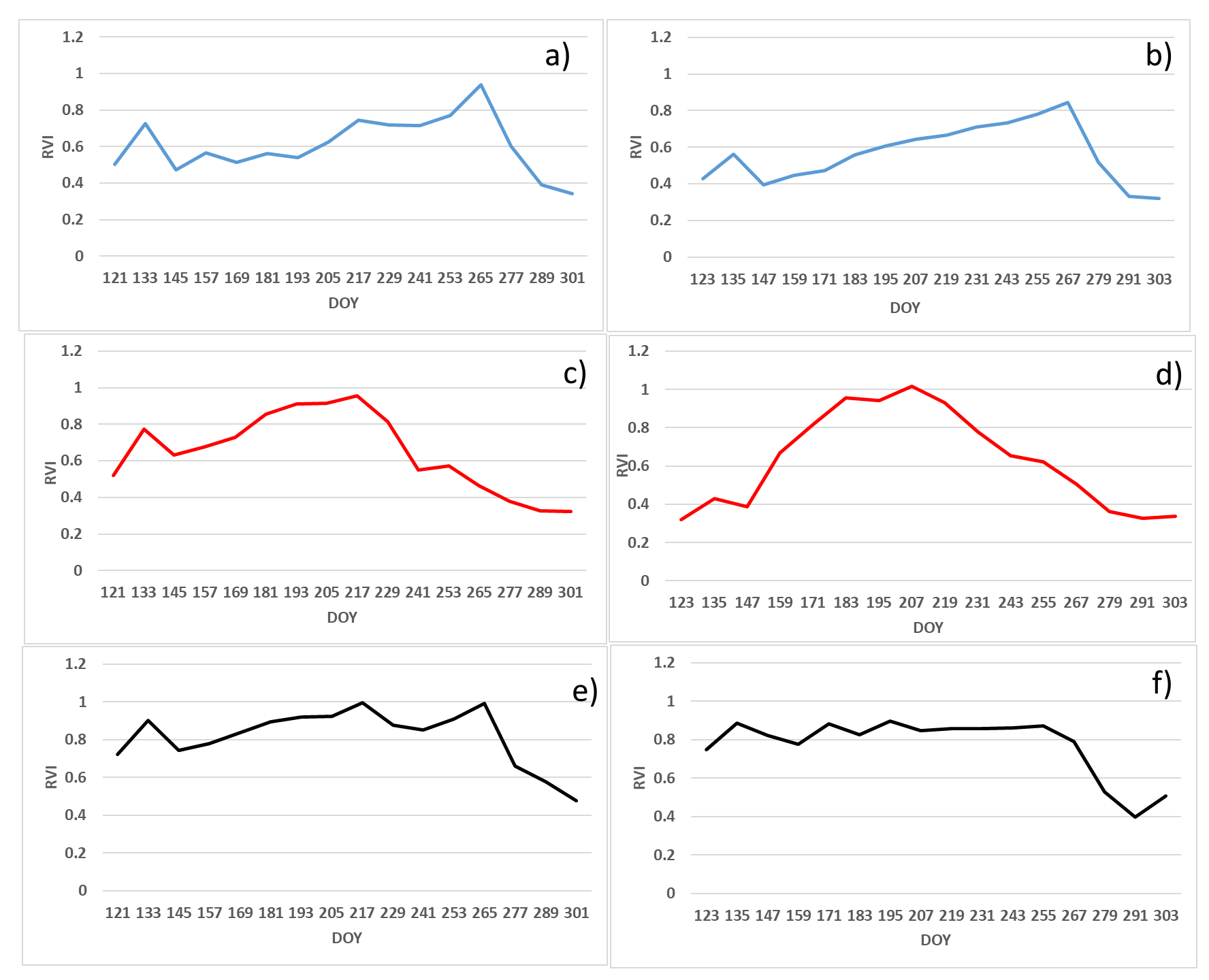

Supplement: Supplementary file 1 [file sensors-23-07902-s001.zip › Figure S1.png]

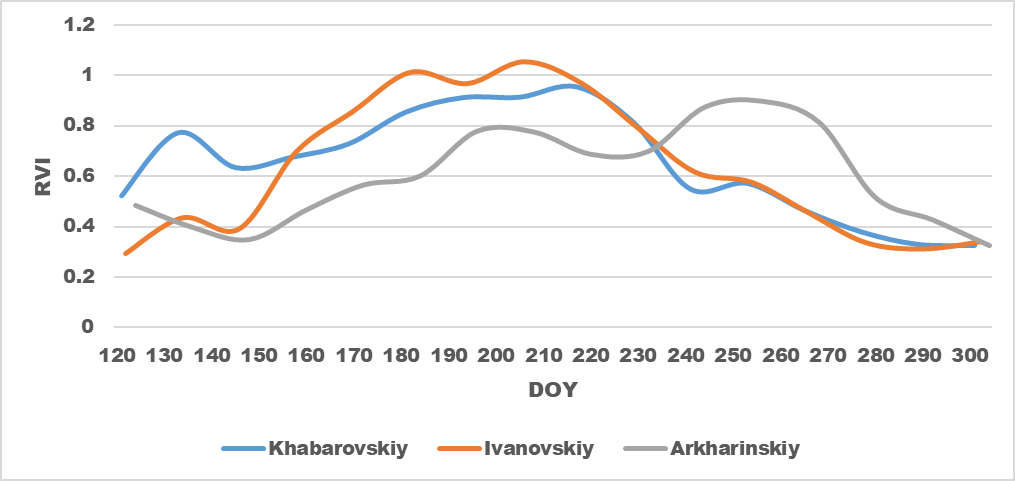

Supplement: Supplementary file 1 [file sensors-23-07902-s001.zip › Figure S2.png]
